# Immunogenicity of COVID-19 Vaccination in Immunocompromised Patients (Auto-COVID-VACC): Protocol for Multicenter Prospective Noninterventional Study

**DOI:** 10.2196/60675

**Published:** 2025-05-26

**Authors:** Louise Marie Cremer, Ullrich Bethe, Peter Borchmann, Veronica Di Cristanziano, Lutz Gieselmann, Sarah Grimm, Martin Hellmich, Julia Jakobs, Julia A Nacov, Julia M Neuhann, Juergen Prattes, Christoph Scheid, Rosanne Sprute, Gertrud Steger, Jannik Stemler, Sibylle C Mellinghoff, Oliver A Cornely

**Affiliations:** 1 Institute of Translational Research, Cologne Excellence Cluster on Cellular Stress Responses in Aging-Associated Diseases (CECAD) Faculty of Medicine and University Hospital Cologne University of Cologne Cologne Germany; 2 Department I of Internal Medicine, Center for Integrated Oncology Aachen Bonn Cologne Duesseldorf (CIO ABCD) and Excellence Center for Medical Mycology (ECMM) Faculty of Medicine and University Hospital Cologne University of Cologne Cologne Germany; 3 German Centre for Infection Research (DZIF), Partner Site Bonn-Cologne Cologne Germany; 4 Institute of Virology Faculty of Medicine and University Hospital Cologne University of Cologne Cologne Germany; 5 Institute of Medical Statistics, Informatics and Epidemiology University of Cologne Cologne Germany; 6 Department of Internal Medicine Division of Infectious Diseases, Excellence Center for Medical Mycology (ECMM) Medical University of Graz Graz Austria; 7 Clinical Trials Centre Cologne (ZKS Köln) Faculty of Medicine and University Hospital Cologne University of Cologne Cologne Germany

**Keywords:** COVID-19, SARS-CoV-2, vaccination, vaccine, infectious diseases, immunocompromised, immunosuppression, communicable disease, observational study

## Abstract

**Background:**

Despite the availability of vaccines, immunocompromised patients are still at high risk for severe COVID-19. Vaccination has been proven to be an effective measure in preventing severe SARS-CoV-2 infections; however, data on B- and T-cell responses are lacking. While vaccination schedules for the general population have been defined, achieving immunogenicity in patients who are immunocompromised remains a challenge.

**Objective:**

The primary objective is to analyze anti-spike–immunoglobulin G (IgG) titers after repeated messenger ribonucleic acid vaccinations in patients who are immunocompromised. Further objectives are to analyze data on humoral immune responses and to evaluate data on cellular immune responses.

**Methods:**

This multicenter, prospective, noninterventional study aims to determine the immunogenicity and reactogenicity of an implemented standard-of-care COVID-19 vaccination strategy in patients who are immunocompromised. A total of 100 patients will be recruited at three study sites. Patients are eligible for study inclusion when they are 18 years or older, vaccinated according to the recent version of the COVID-19 vaccination standard, and if the patient is immunocompromised according to stage 3 of the classification “Stages of Immunosuppression.” The study analyzes B- and T-cell responses generated within the standard-of-care COVID-19 vaccination strategy. Additional blood samples will be drawn at each scheduled outpatient visit. Study-related blood samples will be used to extract ethylenediaminetetraacetic acid plasma and peripheral blood mononuclear cells for evaluation of B- and T-cell responses to COVID-19 vaccinations. For this study, no additional visits or invasive procedures will be performed in addition to standard care.

**Results:**

As of August 2024, the study has enrolled 32 patients. The recruitment phase is still ongoing.

**Conclusions:**

Results will be used to optimize vaccination and booster schedules for patients who are immunocompromised and to increase rates of protection against severe SARS-CoV-2 infections. Further, results may identify risk and treatment factors, which lead to low immune responses in patients vaccinated against COVID-19, as well as the impact of repeated vaccination on B- and T-cell responses.

**Trial Registration:**

ClinicalTrials.gov NCT05597761; https://clinicaltrials.gov/study/NCT05597761

**International Registered Report Identifier (IRRID):**

DERR1-10.2196/60675

## Introduction

The novel SARS-CoV-2 was identified in December 2019 as a cause of severe pneumonia, acute respiratory distress syndrome, and potential multiorgan failure in humans [1,2]. The disease known as COVID-19 has turned into a worldwide pandemic, causing substantial morbidity and mortality, and is now (2024) considered an endemic disease. As of December 15, 2024, the World Health Organization reports a total of 7,079,587 COVID-19 deaths worldwide [3]. Patients who are immunocompromised, such as those with hematological malignancies, are highly affected by SARS-CoV-2 infections due to the high risk of severe courses of infection [4-8]. COVID-19 may not only result in a delay or discontinuation of the treatment for the underlying diseases but also result in high mortality rates of above 30% [4-6,9,10]. Prevention strategies are therefore focused on avoiding SARS-CoV-2 infection in these vulnerable patients and reducing the risk of critical disease following infection. This is addressed by nonpharmacological and pharmacological interventions. Nonpharmacological interventions such as wearing masks, physical distancing, and respiratory hygiene are effective for reducing SARS-CoV-2 transmission but were withdrawn for the general population by state authorities as of June 2022. For example, masking in public is no longer mandatory in many countries. Pharmacological interventions included several antiviral drugs as well as various therapeutic monoclonal antibodies that lost neutralizing capacity with the emergence of Omicron variants [11,12]. At the end of 2020 and the beginning of 2021, the first messenger RNA vaccines against COVID-19 were proven to be effective in the prevention of severe SARS-CoV-2 infection and licensed for use [13-15]. The German Standing Committee on Vaccination of the Robert Koch Institute (RKI) has been recommending 3 antigen contacts (infection or vaccination) to complete basic immunization, of which at least one should be by vaccination. Annual booster recommendations are advised for groups at high risk, such as people with an impaired immune response [16]. COVID-19 vaccination, therefore, represents the mainstay of COVID-19 prevention. Efficacy and safety have been demonstrated for several vaccines, leading to currently 15 vaccines listed by the Paul Ehrlich Institute in Germany and with marketing authorization in Europe (as of October 2024) [17]. Studies have shown that COVID-19 vaccination is efficient in patients who are immunocompromised. However, lower levels of antibodies have been reported in this patient group [18,19]. Patients who are immunocompromised had been excluded from the majority of registered studies. Further, studies focused mainly on B-cell immune responses, resulting in uncertainty regarding the extent of immune response and the role of T cell–generated immune response in these patients. The aim of this study is to determine the immunogenicity and reactogenicity of an implemented standard-of-care (SOC) COVID-19 vaccination strategy (Multimedia Appendix 1) in patients who are immunocompromised [20].

## Methods

### Overview

A new SOC vaccination strategy was implemented for patients who are immunocompromised at 3 university hospitals in Germany, the University Hospital of Aachen, Cologne, and Essen. This approach targets a vaccination strategy that is guided by individual immune responses to COVID-19 vaccines [20] (Multimedia Appendix 1).

### Study Design

This is a multicenter, prospective, noninterventional study conducted at university hospitals in Germany that have implemented the SOC COVID-19 vaccination for patients who are immunocompromised [20]. This study aims to analyze the humoral and cellular immune response data generated within the procedures of the SOC COVID-19 vaccination [20]. Patients who are immunocompromised and will be vaccinated according to the vaccination standard [20] are eligible for study inclusion. To define immunocompromised, the classification “Stages of Immunosuppression” [21] was considered (Multimedia Appendix 2). Patients in stage 3 (eg, patients with a hemato-oncological underlying disease or patients receiving biologicals) are eligible for study inclusion. At each vaccination visit and follow-up visit, both defined in the vaccination standard, enrolled patients will undergo study-related assessment in addition to routinely performed assessments and interventions within their standard care. At the first visit, a SARS-CoV-2 antigen test will be performed. In case of a positive test result, the patient will be excluded from study enrollment. According to the vaccination standard [20], blood draws will be performed at each visit. For study purposes, additional blood samples will be drawn by using the same vein puncture at these visits (see visits and study-related assessments). These samples will be used for additional analysis of the immune response. The samples will be collected and stored until the analysis, which will be performed after the recruitment period. This guarantees that results obtained from study-related immune response assessments do not affect the standard vaccination strategy. Patients will also receive a patient diary before the first vaccination to document adverse drug reactions (ADRs) to the COVID-19 vaccine.

### Study-Related Tests

Peripheral blood mononuclear cells will be isolated from whole blood ethylenediaminetetraacetic acid–containing tubes using density-based separation with Ficoll. Cells will be cryopreserved for immune phenotyping by flow cytometry at the end of the study. Immune phenotyping of lymphocytes allows specific analyses of lymphocytic subsets, which are defined by a higher number and composition of surface antigens. Using a customized panel of antibodies, we will be able to detect the major subsets of B- and T-cell lymphocyte lineages. All study-related immune response measurements will be performed in batches from stored samples after last-patient-last-visit at the University Hospital of Cologne. Sample remains are disposed of after study completion.

### Objectives

Data for primary and secondary objectives are generated within the routine procedures of SOC COVID-19 vaccination [20]. Data for exploratory objectives will be generated from study-related assessments based on study-related blood sampling. Study-related blood sampling will be performed at the same timepoints and from the same vein puncture as for routine standard procedures (Textbox 1).

**Primary, secondary, and explorative objectives.**

**Primary objective**

- To analyze the available data on anti-spike–immunoglobulin G titers after repeated messenger RNA (mRNA) vaccinations in immunocompromised patients

**Secondary objectives**

- To analyze the available data on neutralizing capacity against the Omicron-BA.1 variant after repeated mRNA vaccinations in immunocompromised patients
- To analyze the available data on neutralizing capacity against other Omicron sublineages after repeated mRNA vaccinations in immunocompromised patients
- To analyze the available data on binding antibody response against Omicron variants after repeated mRNA vaccinations in immunocompromised patients
- To analyze the available data on the waning of the humoral immune response against Omicron variants after the last mRNA vaccination in successfully vaccinated patients according to the standard of care
- To analyze reactogenicity data (see solicited adverse events) collected within this study

**Explorative objectives**

- To evaluate B- and T-cell immune response against Omicron variants after repeated mRNA vaccinations in immunocompromised patients
- To evaluate the waning of B- and T-cell immune response against SARS-CoV-2 (in particular Omicron variants) after the last mRNA vaccination in successfully vaccinated patients according to the standard of care
- To evaluate memory B-cell response against the Omicron variant after the last mRNA vaccine, in successfully vaccinated patients

### End Points

The end points are listed in Textbox 2.

**Primary, secondary, and exploratory endpoints.**

**Primary end point**

- Time to reach an adequate immune response according to the recent version of the COVID-19 vaccination standard-of-care [20] after repeated messenger RNA (mRNA) vaccinations (Multimedia Appendices 1 and 3)

**Secondary end points**

- Time to Omicron BA.1-specific neutralizing antibody ID_50_ (50% inhibitory dilution) titers ≥30 after repeated mRNA vaccinations in immunocompromised patients
- Time to anti-spike-1/2 IgG (immunoglobulin G) increase ≥33.8 binding antibody units (BAU)/mL measured by SARS-CoV-2 TrimericS IgG (DiaSorin) after repeated mRNA vaccinations in immunocompromised patients
- Time to decrease of Omicron BA.1–specific neutralizing antibody ID_50_ titers <30 after last mRNA vaccination in successfully vaccinated patients according to the standard-of-care
- Time to anti-spike-1/2 IgG decrease <33.8 BAU/mL after the last mRNA vaccination in successfully vaccinated patients according to the standard-of-care measured by SARS-CoV-2 TrimericS IgG

Anti-spike-1/2 IgG titer ≥ 33.8 BAU/mL was interpreted as reactive, according to the manufacturer’s instructions (DiaSorin). To determine Omicron BA.1-specific neutralizing serum titers, the lowest tested serum dilution was 1:30, which served as the lower limit of quantification.

**Exploratory end points**

- Time to detection of SARS-CoV-2–specific T-cell immunity
- Time to detection of SARS-CoV-2–specific antibody-secreting plasma cells
- Time to detection of SARS-CoV-2–specific memory B cells
- Time to waning of SARS-CoV-2–specific T cellular immunity
- Time to waning of SARS-CoV-2–specific antibody-secreting plasma cells
- Time to waning of SARS-CoV-2–specific memory B cells

### Study Cohort

Eligibility criteria and other details have been listed in Textbox 3.

**Eligibility criteria, premature study withdrawal, and enrollment failure**

**Inclusion criteria**

- Patient is vaccinated according to the recent version of the COVID-19 vaccination standard for immunocompromised patients [20]
- Patient is 18 years of age or older at enrollment
- Written informed consent from patient has been obtained prior to any study-related procedures (see obtaining informed consent)
- Patient is immunocompromised according to stage 3 classification “Stages of Immunosuppression” [21] (Multimedia Appendix 2)

**Exclusion criteria**

- Patient is not vaccinated according to the recent version of the COVID-19 vaccination standard [20]
- Patient has a positive SARS-CoV-2 antigen test at the first visit

**Premature withdrawal from the study**

In case a patient fulfills one of the following criteria, the patient must be withdrawn from the study

- Patient has reached an adequate immune response as defined in the recent version of the COVID-19 vaccination standard [20] at the first visit

In case a patient has to withdraw prematurely from the study, all data that have been collected between enrollment and withdrawal will be used for the analysis.

**Enrollment failure**

Enrollment failures are not expected as patients, who are willing to participate and are vaccinated according to the recent version of the COVID-19 vaccination standard [20], are all included in the study.

A minimum set of enrollment failure information is required. Minimum information includes date of informed consent, demography, enrollment failure details, eligibility criteria, and information on any solicited adverse events or serious adverse events that may have occurred from the time informed consent was obtained to the time of withdrawal.

### Visits and Study-Related Assessments

Visit details are listed in Textbox 4 and Table 1. The visit schedule is displayed in Table 1.

**Visits and study-related assessments**

**Visit 1**

Following assessments will be performed at this visit:

- Review of inclusion and exclusion criteria
- Written informed consent will be obtained
- Demographics including age and sex will be obtained
- Relevant medical history including all prior chemotherapy regimens and radiation therapy will be collected
- Collection and review of previous and concomitant medication
- SARS-CoV-2 antigen test obtained from nasal, pharyngeal, nasopharyngeal, or oropharyngeal swab
- Patient diary will be handed out and explained to the patient. Patient is instructed to record solicited adverse events that occur within 7 days after the time of vaccination in the patient diary (the day of vaccination plus the following 6 days)
- Prevaccination blood sampling for SARS-CoV-2–specific B- and T-cell immune response assessment will be performed (maximum 36 mL blood)

**All following visits**

- Review of any changes of concomitant medication since the last visit and documentation in the electronic case report form if applicable
- Review of patient diary and documentation of adverse events (see safety)
- Prevaccination blood sampling for SARS-CoV-2–specific B- and T-cell immune response assessment will be performed (maximal 18 mL blood)

**Follow-up visits**

Days in the follow-up period are counted from the day of the last vaccination onward. The visit schedule is displayed in Table 1.

**Follow-up visit 1**

The following assessments will be performed at this visit:

- Collection of patient diary and documentation of adverse events (see Safety section for details). The patient diary will remain at the study site together with the patients’ medical records
- Blood sampling for SARS-CoV-2–specific B- and T-cell immune response assessment (maximal 18 mL blood)

**All following follow-up visits**

The following assessment will be performed at all subsequent follow-up visit:

- Blood sampling for SARS-CoV-2–specific B- and T-cell immune response assessment (maximal 18 mL blood)
- Previous and concomitant medication of interest

Information on previous and concomitant immunosuppressive medication within 90 days prior to providing informed consent will be recorded in the electronic case report form. In addition, all COVID-19 vaccinations and all therapeutic neutralizing antibodies against SARS-CoV-2 (tixagevimab-cilgavimab, sotrovimab, convalescent plasma, etc) a patient has ever received will be documented in the electronic case report form.

**Table 1:** Visit schedule.

Procedures	Vaccination period		Follow-up period	
	Visit 1	All following visits^a^	FU^b^ 1	All following FUs
Day^c^	0^c^ (42^d^)	28-day intervals^c,d^	28^e^	28-day intervals^e^
Visit window (days)	±5	±5	±5	±5
Informed consent	✓			
Demographics	✓			
Review of inclusion and exclusion criteria	✓			
Medical history	✓			
Concomitant medication	✓	✓	✓	✓
SARS-CoV-2 antigen test	✓			
SARS-CoV-2–specific B- and T-cell immune response^f^	✓	✓	✓	✓
Dispensing of the study diary	✓			
Collection of the study diary			✓	
Review of diary and documentation of solicited adverse events^g^		✓	✓	
Complete case report form	✓	✓	✓	✓
Standard cellular immunity^h,i^	✓	✓	✓	✓
SARS-CoV-2 Quantiferon^i^	✓	✓	✓	✓
Omicron-BA.1–specific nAB^i,j,k^	✓	✓	✓	✓
Anti-spike-1/2 IgG^l^ antibodies^i,j^	✓	✓	✓	✓
Anti-nucleocapsid IgG^l^ antibodies^i^	✓	✓	✓	✓
Anti-SARS-CoV-2 vaccination^i^	✓	✓		

^a^Visit only in case anti-spike-1/2-IgG titer was <847 BAU (binding antibody units)/mL at the previous visit according to standard-of-care COVID-19 vaccination.

^b^FU: follow-up.

^c^Day 0 is the day of the first visit and first vaccination. All days in the vaccination phase are counted in relation to the last vaccination, respectively.

^d^Applicable for patients posthematopoietic stem cell transplantation (HSCT) or chimeric antigen receptor (CAR)–T cell treatment. Day 0 is considered the day of HSCT or CAR-T cell treatment. All days in the vaccination phase are counted in relation to the day of HSCT or CAR-T cell treatment, respectively.

^e^Days in the follow-up phase are always counted in relation to the last vaccination.

^f^Maximum 36 mL blood is collected at visit 1. At all other visits, maximum 18 mL blood is collected.

^g^Solicited adverse events will be documented for 7 days after each vaccination.

^h^Contains blood cell count, T-lymphocytes (CD3, CD4, CD8), natural killer cells (CD16+56), B-lymphocytes (CD19), and activated T cells (CD38, HLA-DR).

^i^Performed as part of the COVID-19 vaccination standard [20] in the clinical routine. Data generated within the standard procedures will be used for analysis of the study objectives.

^j^Anti-spike-1/2 IgG titer ≥33.8 BAU/mL was interpreted as reactive, according to the manufacturer’s instructions (DiaSorin). To determine Omicron BA.1-specific neutralizing serum titers, the lowest tested serum dilution was 1:30, which served as the lower limit of quantification.

^k^nAB: neutralizing antibody.

^l^IgG: immunoglobulin G.

### Safety

#### Solicited Adverse Events

In order to evaluate the reactogenicity of COVID-19 vaccinations applied within the SOC [20], this study collects solicited adverse events (AEs) for 7 days (the day of vaccination plus the following 6 days) after each vaccination. As a noninterventional study, this study focuses on solicited AEs only, that is, on a subset of all AEs. Solicited AEs will be collected by the patient using a patient diary. Solicited AEs will be documented starting on the day of each vaccination within the SOC [20] and the following 6 days. The grading system of solicited AEs is based on the Toxicity Grading Scale for Healthy Adult and Adolescent Volunteers Enrolled in Preventive Vaccine Clinical Trials [22] and the Common Terminology Criteria for Adverse Events v5.0 (CTCAE) [23] grading systems (Multimedia Appendix 2).

#### Adverse Drug Reactions

An ADR is defined as a response to a drug that is noxious and unintended and which occurs at doses normally used in humans for prophylaxis, diagnosis, or therapy of disease or modification of physiological function. All ADRs related to one of the COVID-19 vaccines used within the COVID-19 vaccination standard [20] occurring during the study period will be documented in the electronic case report form (eCRF). A serious AE or serious ADR is any untoward medical occurrence that at any dose meets any of the following criteria: (1) results in death, (2) is life-threatening, (3) requires inpatient hospitalization or prolongation of existing hospitalization, (4) results in persistent or significant disability or incapacity, or (5) is a congenital anomaly or birth defect. Serious AEs occurring 7 days (the day of vaccination plus the following 6 days) after each vaccination will be collected in the eCRF.

Serious ADRs occurring during the study period (including the follow-up period) will also be collected in the eCRF.

### Monitoring

The study sites will be monitored to ensure the quality of the data collected. The objectives of the monitoring procedures are to ensure that the subject’s safety and rights as a study participant are respected; that accurate, valid, and complete data are collected; and that the study is conducted in accordance with the study protocol, the principles of Good Clinical Practice (GCP), and local legislation. The exact extent of the monitoring procedures is described in a separate monitoring plan. Monitoring will follow a risk-adapted on-site monitoring strategy. All principal investigators (PIs) agree that the monitor regularly visits the study site and assures that the monitor will receive appropriate support in their activities at the study site, as agreed in separate contracts with each study site. The declaration of informed consent includes a statement allowing the monitor to compare the CRFs with the study subject’s medical records (doctor’s notes, laboratory printouts, etc). The PI will secure access for the monitor to all necessary documentation for study-related monitoring. A monitoring visit report is prepared for each visit describing the progress of the study and any challenges. The PI will reasonably consider the corrective and preventive measures suggested by the monitor. All participant data relating to the study will be recorded on eCRFs. The investigator is responsible for verifying that data entries are accurate and correct by electronically signing the CRF. The investigator must permit study-related monitoring, audits, Research Ethics Committee or National Competent Authority review, and regulatory agency inspections, and provide direct access to source data documents.

### Documentation

PIs will oversee and coordinate data collection, entry, and protection. Study-specific data will be collected by the study staff using designated source documents. Standard GCPs will be followed to ensure accurate, reliable, and consistent data collection. All study data must be verifiable to the source documentation. All source documents will be kept in a locked facility at the study site.

### Data Management

Data management activities will be conducted by the Clinical Trials Centre Cologne (CTCC). The IT infrastructure and data management staff will be provided by the CTCC. The study database will be developed and validated before data entry based on standard operating procedures at the CTCC. The data management system is based on commercial trial software and stores the data in a database. All changes made to the data are documented in an audit trail. The trial software has a user and role concept that can be adjusted on a trial-specific basis. The database is integrated into a general IT infrastructure and safety concept with a firewall and backup system. The data are backed up daily. After completion and cleaning of data, the database is locked and the data are exported for statistical analysis. The data will be entered online at the study sites via the internet. Plausibility checks are run during data entry, thereby detecting many discrepancies immediately. The CTCC data management will conduct further checks for completeness and plausibility and will clarify any questions with the study sites electronically via the trial software. These electronic queries have to be answered by the study site without unreasonable delay. Further details will be specified in the data management manual. A guidance document for data entry in the eCRF will be provided. All participant data relating to the study will be recorded on eCRFs unless transmitted to the sponsor or designee electronically (eg, laboratory data) via secure platform transfer or encrypted email. The investigator is responsible for verifying that data entries are accurate and correct by electronically signing the CRF.

### Archiving

All essential documents according to GCP of the International Council for Harmonisation of Technical Requirements for Registration of Pharmaceuticals for Human Use (ICH-GCP) chapter 8 will be archived for at least 10 years unless longer times are foreseen by local regulatory requirements. No records may be destroyed during the retention period without the written approval of the lead PI of the investigator-initiated trial. No records may be transferred to another location or party without written notification to the lead PI of the investigator-initiated trial. Digitization of paper-based documentation must meet regulatory requirements, especially concerning quality assurance, completeness, and correctness. Paper-based documentation must not be destroyed after digitization and must be archived according to applicable regulations.

### Timelines for the Study Participant

Within a SOC vaccination [20], every patient will receive a varying number of COVID-19 vaccinations. Accordingly, patients will be documented within this study for varying durations depending on the number of vaccine doses needed according to the recent version of the COVID-19 vaccination standard [20] (Table 2).

**Table 2:** Study timeline.

Milestone	Timepoint (quarter)
First patient first visit	Q2 2023
Last patient first visit	Q3 2024
Last patient last visit	Q3 2024
End of study	Q4 2024
Final study report	Q4 2024

### Statistical Considerations

The primary analysis set comprises all enrolled patients who received at least one vaccination within the COVID-19 vaccination standard [20]. The statistical analysis is essentially descriptive and focuses on estimation of the time to event, that is, primary and secondary end points. The event-time distributions are described by the Kaplan-Meier method [24] with pointwise 95% confidence bounds [25] (18, “log-log”). Relevant percentiles (eg, quartiles) with 95% CIs are derived. Generally, quantitative variables are summarized by mean, SD, and percentiles (0, 25, 50, 75, 100), and qualitative variables by absolute (ie, count) and relative (ie, percentage) frequencies. Extreme values (“outliers”) are flagged and listed.

### Sample Size

The target sample size of 100 is sufficient to estimate event-free probabilities around the median “survival” with acceptable precision (half-width of a 95% CI) of about 10% for censoring rates less than 20% (Table 3).

**Table 3:** Sample size calculation.

Number of events	Half width^a^ (%)
100	9.7
90	9.9
80	10.2
70	10.6
60	11.1
50	11.8
40	13.3
30	15.4
20	19.7
10	21.7
0	—^b^

^a^“Absolute” percentage points.

^b^Not applicable.

### Ethical Considerations

#### Ethics

This study will be conducted in accordance with the protocol and with the following:

- Consensus on ethical principles derived from international guidelines, including the Declaration of Helsinki and the Council for International Organizations of Medical Sciences international ethical guidelines
- Applicable ICH-GCP guidelines dated July 1996 and its Addendum E6(R2) of June 2017

The study was approved by the lead Ethics Committee of the University of Cologne (Cologne, Germany; 22-1279-NIS), and approval by the responsible Ethics Committee for the study sites Aachen and Essen followed.

#### Obtaining Informed Consent

Patients will be informed verbally and in writing in a comprehensible language about the nature, scope, and possible consequences by the PI or subinvestigator. This includes consent to data access by representatives of the lead PI of the Investigator Initiated Trial (eg, monitors). The patient will be informed of potential benefits and possible risks associated with participation in the study. Of note, risks associated with study participation are considered minimal and are limited to data protection. No additional interventions (eg, vein puncture) or treatments are foreseen as part of study participation. Patients will be informed that withdrawal of consent is possible at any time without giving a reason and without jeopardizing the patients’ further course of treatment. It is the PIs or subinvestigators responsibility to ensure that the patient fully understands the implications of participation in the study. Patients must not be enrolled in the study unless they have consented to take part in writing. The patients’ medical records must contain a statement that written informed consent was obtained, including the date on which the consent was obtained. In addition, the authorized person at the study site (investigator or subinvestigator) who obtains the informed consent must also sign the informed consent form (ICF) according to the ICH-GCP guidelines. Patients must be reconsented to the most current version of the ICFs during their participation in the study if relevant. The signed consent form is archived at the study site as the original. Patients receive copies of the signed ICF. The ICF and all other documents handed out to the study participants will be submitted to the ethics committee before use. Monitors will check that the most recent ICF was used before the patient’s inclusion and that it was dated and signed by the patient himself or herself.

## Results

As of August 2024, the Auto-COVID-VACC study has enrolled 32 patients. The study is still in the recruitment phase.

## Discussion

### Overview

The COVID-19 vaccination standard, implemented at the University Hospital of Aachen, Cologne, and Essen, poses an individual vaccination approach for each patient depending on their individual immune response. The evaluation of the data on humoral and cellular immune responses generated within this COVID-19 vaccination standard will be used to optimize vaccination schedules for patients who are immunocompromised. Results may further help to identify the optimal timing of booster vaccinations within this patient population and their subsets and will lead to increased rates of protection against severe SARS-CoV-2 infections. As the patients included in this study have varying underlying diseases (eg, multiple myeloma, Hodgkin lymphoma) and follow different treatment concepts (eg, chemotherapy, immunotherapy, hematopoietic stem cell transplantation), study results may identify risk and treatment factors on the humoral and cellular immune response in patients receiving COVID-19 vaccination.

### Limitations

This study design has several a priori limitations. The study focuses on COVID-19 vaccination only. Patients with SARS-CoV-2 infection are excluded when tested positive; however, studies have shown that SARS-CoV-2 infections also induce immune responses and therefore increase protection [26]. Future study results have to be interpreted cautiously due to the potential risk of bias as data are collected as part of an observational study and in the frame of a SOC COVID-19 vaccination schedule. Patients therefore are not randomized. Further, potential confounders are not included in the statistical analysis.

### Conclusions

Patients who are immunocompromised are at high risk for severe SARS-CoV-2 infections. Data on the efficacy of COVID-19 vaccination in this group are still lacking. The study aims to determine the immunogenicity and reactogenicity of the COVID-19 vaccination standard in patients who are immunocompromised by evaluating the available data on humoral and cellular immune responses.

## Appendix

Multimedia Appendix 1 COVID-19 vaccination standard.

Multimedia Appendix 2 Stages of immunosuppression (Table S1); Definition and grading of solicited adverse events (Table S2).

Multimedia Appendix 3 Primary end point definition for the Auto-COVID-VACC trial.

## Abbreviations

ADR: adverse drug reaction
AE: adverse event
CAR: chimeric antigen receptor
CTCAE: Common Terminology Criteria for Adverse Events
CTCC: Clinical Trials Centre Cologne
eCRF: electronic case report form
ICF: informed consent form
ICH-GCP: Good Clinical Practice (of the International Council for Harmonisation of Technical Requirements for Pharmaceuticals for Human Use)
IgG: immunoglobulin G
PI: principal investigator
SOC: standard of care
