# Supplementary material for: Immunogenicity of COVID-19 Vaccination in Immunocompromised Patients (Auto-COVID-VACC): Protocol for Multicenter Prospective Noninterventional Study
Source: JMIR Res Protoc. 2025 May 26;14:e60675. doi: 10.2196/60675 (PMC12149776; doi:10.2196/60675)
Supplement: Multimedia Appendix 1 [file resprot_v14i1e60675_app1.pdf]

Form - Clinical standard - COVID-19 vaccination schedule for immunocompromised patients with an anti-spike-IgG titre <847 BAU/ml *(English translation)*

Authors: Univ. Prof. Dr. Oliver A. Cornely, Prof. Dr. Dr. h.c. Christoph Scheid, Prof. Dr. Peter Borchmann
Date: 04/03/2024

1. Background

The immune response and therefore the protective effect of COVID-19 vaccination is highly variable in individuals. In particular, this depends on underlying diseases and therapies, which can reduce the immune response following vaccination. Immunocompromised people therefore require a structured, individualised approach to vaccination in order to achieve best possible protection against COVID-19 at an early stage.

2. Purpose

This standard-of-care (SOC) represents a COVID-19 vaccination schedule for immunocompromised patients with an anti-spike-IgG titre <847 BAU/ml. It is an individualised, antibody-controlled approach to reach immunity against COVID-19.

3. Procedure

The following SOC COVID-19 vaccination schedule should be considered for all immunocompromised patients with an anti-spike-IgG titre <847 BAU/ml. To define immunocompromised, the classification of Wiedermann et al. (2016) “Stages of immunosuppression” should be considered[1].
This SOC vaccination schedule should be considered regardless of whether the patient has already received COVID-19 vaccination or has been infected with SARS-CoV-2.
Anti-spike-IgG titres should be routinely measured upon hospital admission.
Immunocompromised patients receive up to 8 COVID-19 vaccinations. Patients after autologous or allogeneic haematopoietic stem cell therapy (HSCT) or after CAR-T cell therapy receive a minimum of three COVID-19 vaccinations. The exact number of vaccinations depends on the patient’s individual immune response (Anti-spike-IgG titre).
This SOC should not to be used for the following patients:

- Patients with confirmed SARS-CoV-2 infection in the last 3 months before receiving the first vaccination within the SOC
- Patients, who have already been vaccinated against COVID-19 outside of this SOC
- Patients with a medical contraindication for COVID-19 vaccination with a mRNA vaccine
- Patients with neutropenia

3.1 COVID-19 vaccination schedule for immunocompromised patients with an anti-spike-IgG titre <847 BAU/ml

Generally, mRNA vaccines are recommended due to their higher immunogenicity.
According to the recommendations of the Robert-Koch-Institute (RKI), an authorised and by the WHO recommended variant-adapted mRNA vaccine should be used to achieve basic immunisation (3 antigen contacts).

Form - Clinical standard - COVID-19 vaccination schedule for immunocompromised patients with an anti-spike-IgG titre <847 BAU/ml *(English translation)*

The first COVID-19 vaccination should be administered earliest 28 days after the last vaccination. For patients after autologous or allogeneic HSCT or after CAR-T cell therapy, the first COVID-19 vaccination is recommended 42 days (±5) after HSCT or CAR-T cell therapy. Vaccinations against COVID-19 should not be administered on the same day as chemotherapy or in patients with neutropenia. All patients after autologous or allogeneic HSCT or after CAR-T cell therapy receive at least three COVID-19 vaccinations. The first three COVID-19 vaccinations should be administered regardless of the antibody titres. If an adequate concentration of anti-spike-IgG cannot be detected in serum after the third COVID-19 vaccination, further vaccinations are recommended.

In immunocompromised patients, who did not receive cellular therapy, anti-spike-IgG titres in serum are already analysed from the first COVID-19 vaccination onwards and used as a basis for deciding if further COVID-19 vaccinations are required. Vaccine doses should be administered in 28-day (±5) intervals.

An adequate concentration of anti-spike-IgG is defined as a titre ≥847 BAU/ml[3]. If the anti-spike-IgG titre is <847 BAU/ml after a total of 8 COVID-19 vaccinations, no further COVID-19 vaccinations should be administered within this SOC.

The humoral and cellular immune responses are determined before each COVID-19 vaccination (sections 3.3 and 3.4).

If there is no new evidence for the superiority of one or the other adapted mRNA vaccine, it is recommended to carry out the entire vaccination series with the same COVID-19 vaccine (with the exception of any necessary basic immunisation). If a switch from one mRNA vaccine to another vaccine (mRNA or non-mRNA) is indicated due to medical reasons, this is possible.

The SOC COVID-19 vaccination schedule is displayed in Figure 1 and 2.

3.2 Follow-up-Visits

Once patients have reached an anti-spike-IgG titre of ≥847 BAU/ml, no further COVID-19 vaccinations will be administered as part of this SOC. The patient then proceeds to the follow-up phase. The follow-up-phase starts 28 days (±5) after the last COVID-19 vaccination and includes a total of 6 visits in 28-day intervals (Figures 1 and 2). If an adequate concentration of anti-spike-IgG cannot be detected 4 weeks after the 8th COVID-19 vaccination, no further follow-up visits take place.

3.3 Humoral immune response

The following parameters should be determined immediately before each COVID-19 vaccination and at each follow-up examination as part of this SOC:

- Omicron-BA.1-specific neutralising antibodies in serum
- Anti-SARS-CoV-2 S1/2 IgG in serum
- Anti-SARS-CoV-2 nucleocapsid in serum

3.4 Cellular immune response

Even in absence of a detectable humoral immune response, immunity at cellular level can be observed in some people with an underlying haematological disease.

However, the extent to which cellular immunity protects against severe courses of SARS-CoV-2 is not known in detail.
As part of this SOC COVID-19 vaccination schedule for immunocompromised patients with an anti-spike-IgG titre <847 BAU/ml, the following parameters should be determined before each vaccination and at each follow-up examination:

- White blood cell differentiation
- T-lymphocytes (CD3, CD4, CD8)
- NK-cells (CD16+56)
- B-lymphocytes (CD19)
- Activated T-cells (CD38)
- Activated T-cells (HLA-DR)
- SARS-CoV-2 specific Quantiferon®

4. Quality assurance

The COVID-19 pandemic has led to numerous scientific findings over the last four years. As a result, new diagnostic and therapeutic approaches have been developed and implemented within very short time. One of these is the use of mRNA vaccines against COVID-19. The recommendations for the optimal use of COVID-19 vaccinations in the population are a dynamic process and are constantly being revised. For many patient cohorts, the optimal vaccination strategy has not yet been defined. This also includes immunocompromised patients.
Following this SOC COVID-19 vaccination schedule patients are vaccinated under continuous antibody monitoring using a structured, individualised approach. Antibody-guided vaccinations have already been established in practice for diphtheria, tick-borne encephalitis, hepatitis A/B and *Haemophilus influenzae type B* in immunocompromised patients[1]. For COVID-19 vaccination this approach is also recommended by the RKI since January 2024[2]. Controlled COVID-19 immunisation addresses inter-individual differences in the immunocompetence. This should make it possible to protect immunocompromised patients against COVID-19 as early and effective as possible and therefore optimise quality in terms of an optimal, holistic clinical management.
Both humoral and cellular immune parameters are determined in order to enable structured decision-making regarding the number of COVID-19 vaccinations within the scope of this SOC.

Form - Clinical standard - COVID-19 vaccination schedule for immunocompromised patients with an anti-spike-IgG titre <847 BAU/ml *(English translation)*

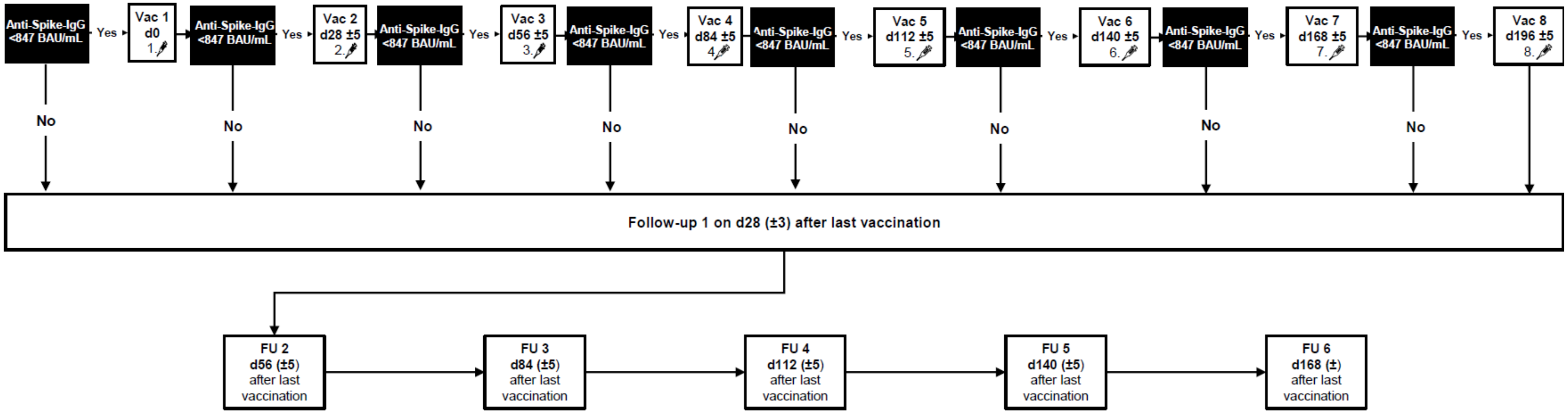

Figure 1 COVID-19 vaccination schedule for immunocompromised patients with anti-spike-IgG <847 BAU/ml

* Blood samples for the determination of the anti-spike-IgG titres are taken immediately before vaccinations 1 to 8 (Vac 1 to Vac 8). The results are then analysed before the next scheduled vaccination. All patients receive up to 8 vaccinations depending on their individual antibody titre. A vaccination is only administered if an anti-spike IgG titre of <847 BAU/ml was detected in the blood sample taken immediately before the last vaccination. A maximum of 8 vaccinations are administered in total within this SOC.
Abbreviations: d = day; FU = follow-up; Vac = COVID-19 vaccination

Form - Clinical standard - COVID-19 vaccination schedule for immunocompromised patients with an anti-spike-IgG titre <847 BAU/ml *(English translation)*

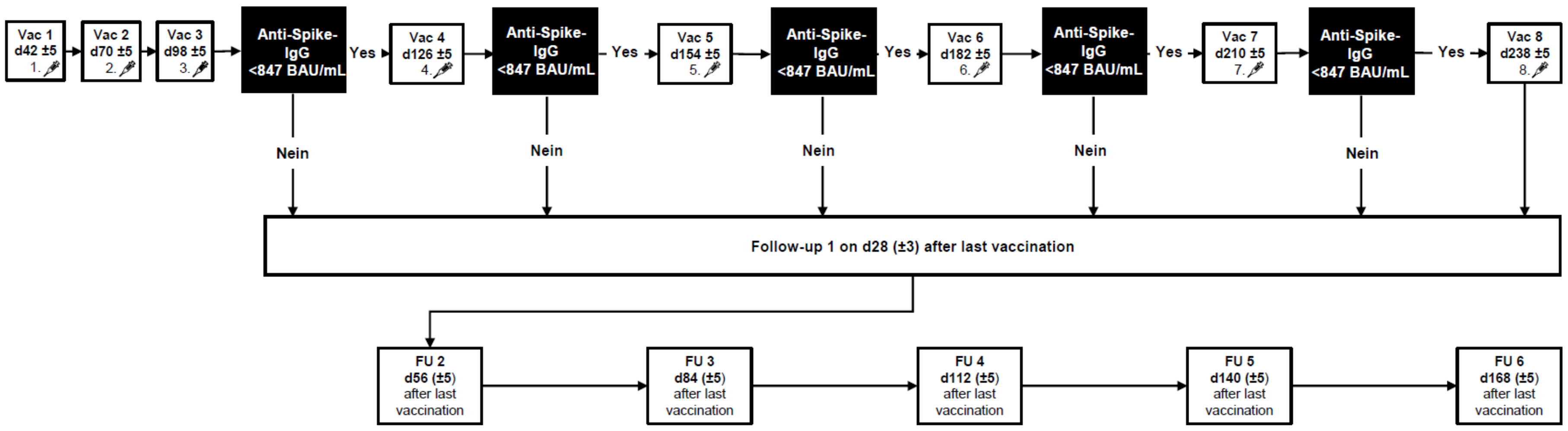

Figure 2 COVID-19 vaccination schedule for immunocompromised patients after cellular therapy with anti-spike-IgG <847 BAU/ml

* Blood samples for the determination of the anti-spike-IgG titres are taken immediately before vaccinations 1 to 8 (Vac 1 to Vac 8). The results are then analysed before the next scheduled vaccination.
All patients receive vaccination 1 to vaccination 3 (Vac 1 to Vac 3). The 4th vaccination is only administered if an anti-spike IgG titre of <847 BAU/ml was detected in the blood sample taken immediately before vaccination 3 (Vac 3). The same procedure applies to vaccination 4 to vaccination 8 (Vac 4 to Vac 8). In total, a maximum of 8 vaccinations are administered under this standard.
Abbreviations: d = day; FU = follow-up; Vac = COVID-19 vaccination

5. References

1. Wiedermann U, Sitte HH, Burgmann H, et al. Impfungen bei Immundefekten/Immunsuppression. Wien Klin Wochenschr 2016;128:337-376.
2. Epidemiologisches Bulletin 04/2024. Robert Koch Institut. (Epidemiologisches Bulletin 4/2024 (rki.de)).
3. Gieselmann L, Di Cristanziano V, Dewald F, Klein F. Primary endpoint definition for the Auto-COVID-VACC trial. 2023.
