# Supplementary material for: Immunogenicity of COVID-19 Vaccination in Immunocompromised Patients (Auto-COVID-VACC): Protocol for Multicenter Prospective Noninterventional Study
Source: JMIR Res Protoc. 2025 May 26;14:e60675. doi: 10.2196/60675 (PMC12149776; doi:10.2196/60675)
Supplement: Multimedia Appendix 2 [file resprot_v14i1e60675_app2.docx]

# Supplementary Appendix 2

## Table S1. Stages of immunosuppression – Stage III *(English translation)*[1]

| HIV-Infection | CD4-cells ≤200/mm^3^/<15% for children |
| --- | --- |
| HSCT | ≤2a or under immunosuppressive therapy or with GvHD |
| SOT | ≤1a or under immunosuppressive therapy or with GvHD |
| Treatment of organ rejection after SOT | |
| Acute hematological disease, metastatic malignant disease | |
| CLL | |
| Recent radiotherapy (at least six weeks after radiotherapy; determination of lymphocyte count) | |
| Aplastic Anaemia | |
| Congenital Immunodeficiency | |
| Cortisone-therapy (Prednisolone or Prednisolone equivalent dose) | >20 mg/day for >2 weeks |
| Transplant-related immunosuppressants | Ciclosporin |
|  | Tacrolimus |
|  | Sirolimus |
|  | Mycophenolat-mofetil |
|  | Mitoxantrone |
| Some chemotherapeutic agents | |
| The majority of biologicals (when interacting with immune cells) | e.g. TNF-α-blocker (Adalimumab, Certolizumab-pegol, Etanercept, Golimumab, Infliximab), Alemtuzumab, Natalizumab, Rituximab etc. |

*HSCT = Haematopoietic Stem Cell Transplantation; GvHD = Graft-versus-Host-Disease; SOT = Solid Organ Transplant; CLL = Chronic Lymphocytic Leukaemia*

## Table S2. Definition and grading of solicited adverse events.

| **Reaction/Symptom** | **Grade 0** | **Grade 1**  **-**  **Mild** | **Grade 2**  **-**  **Moderate** | **Grade 3**  **-**  **Severe** | **Grade 4**  **-**  **Potentially life-threatening** |
| --- | --- | --- | --- | --- | --- |
| **Local AEs (at the site of injection)** | | | | | |
| **Pain** | None | Does not interfere with activity | Repeated use of non-narcotic pain reliever >24 hours or interference with activity | Any use of narcotic pain reliever or prevents daily activity | Requires ER visit or hospitalization |
| **Tenderness** | None | Mild discomfort to touch | Discomfort with movement | Significant discomfort at rest | Requires ER visit or hospitalization |
| **Erythema/Redness/Rash** | <2.5 cm | 2.5 – 5 cm | 5.1 – 10 cm | >10 cm | Necrosis or exfoliative dermatitis |
| **Swelling/Induration/Hardness** | <2.5 cm | 2.5 – 5 cm and does not interfere with activity | 5.1 – 10 cm or interferes with activity | >10 cm or prevents daily activity | Necrosis |
| **Pruritus/Itching*** | None | No interference with activity | Some interference with activity not requiring medical intervention | Prevents daily activity or requires medical intervention* | Requires ER visit or hospitalization |
| **Urticaria** | None | No intervention indicated | Intervention indicated for <24 hours | Intervention indicated for ≥24 hours | Requires ER visit or hospitalization |
| **Systemic AEs** | | | | | |
| Chills | None | Mild sensation of cold; shivering; chattering of teeth | Moderate tremor of the entire body; narcotics indicated | Severe or prolonged, not responsive to narcotics | Requires ER visit or hospitalization |
| Fever (°C)** | <38.0 | 38.0 – 38.4 | 38.5 – 38.9 | 39.0 – 40.0 | >40 |
| Axillary swelling or tenderness ipsilateral to the side of injection | None | No interference with activities | Repeated use of non-narcotic pain reliever >24 hours or interference with activity | Any use of narcotic pain reliever or prevents daily activity | Requires ER visit or hospitalization |
| Pain and/or swelling in the upper extremity ipsilateral to the side of injection | None | No interference with activities | Repeated use of non-narcotic pain reliever >24 hours or interference with activity | Any use of narcotic pain reliever or prevents daily activity | Requires ER visit or hospitalization |
| Fatigue and malaise | None | No interference with normal activities | Some interference with activity | Significant: prevents daily activity | Requires ER visit or hospitalization |
| Myalgia | None | No interference with normal activities | Some interference with activity | Significant: prevents daily activity | Requires ER visit or hospitalization |
| Arthralgia | None | No interference with normal activities | Some interference with activity | Significant: prevents daily activity | Requires ER visit or hospitalization |
| Headache | None | No interference with activities | Repeated use of nonnarcotic pain reliever >24 hours or some interference with activity | Significant; any use of narcotic pain reliever or prevents daily activity | Requires ER visit or hospitalization |
| Dizziness | None | Mild unsteadiness or sensation of movement | Moderate unsteadiness or sensation of movement; limiting instrumental ADL*** | Severe unsteadiness or sensation of movement; limiting self-care ADL**** | Requires ER visit or hospitalization |
| Insomnia | None | Mild difficulty falling asleep, staying asleep or waking up early | Moderate difficulty falling asleep, staying asleep or waking up early | Severe difficulty in falling asleep, staying asleep or waking up early | Disabling |
| Nausea/vomiting | None | No interference with activity or 1 – 2 episodes/24 hours | Some interference with activity or >2 episodes/24 hours | Prevents daily activity, requires outpatient IV hydration | ER visit or hospitalization for hypotensive shock |
| Diarrhea | None | Increase of <4 stools per day over baseline; mild increase in ostomy output compared to baseline | Increase of 4 - 6 stools per day over baseline; moderate increase in ostomy output compared to baseline; limiting instrumental ADL | Increase of ≥7 stools per day over baseline; hospitalization indicated; severe increase in ostomy output compared to baseline; limiting self-care AD | Requires ER visit or hospitalization |
| Generalized maculo-papular rash | None | Macules/papules covering <10% BSA with or without symptoms (e.g., pruritus, burning, tightness) | Macules/papules covering 10 - 30% BSA with or without symptoms (e.g., pruritus, burning, tightness); limiting instrumental ADL; rash covering >30% BSA with or without mild symptoms | Macules/papules covering >30% BSA with moderate or severe symptoms; limiting self-care ADL | Requires ER visit or hospitalization |
| Anorexia | None | Loss of appetite without alteration in eating habits | Oral intake altered without significant weight loss or malnutrition; oral nutritional supplements indicated | Associated with significant weight loss or malnutrition (e.g., inadequate oral caloric and/or fluid intake); IV fluids, tube feedings or TPN indicated | Life-threatening consequences |
| * Over-the-counter topical therapy (e.g.; cooling pads, anti-itching creams, etc.) is not considered as medical intervention.  ** Measured as oral temperature (no recent hot or cold beverages or smoking before taking the temperature), axillary temperature (no recent significant physical activity before taking the temperature) or ear temperature.  *** Instrumental ADL refer to preparing meals, shopping for groceries or clothes, using the telephone, managing money etc.  **** Self-care ADL refer to bathing, dressing and undressing, feeding self, using the toilet, taking medications, and not bedridden.  **Abbreviations:** ADL = activities of daily living; AE = adverse event; BSA = body surface area; ER = emergency room; IV = intravenous; OTC = over-the-counter; TPN = total parenteral nutrition | | | | | |

Reference:

1. Wiedermann, U., et al., *Impfungen bei Immundefekten/Immunsuppression – Expertenstatement und Empfehlungen.* Wiener klinische Wochenschrift, 2016. **128**(4): p. 337-376.
