# Supplementary material for: Immunogenicity of COVID-19 Vaccination in Immunocompromised Patients (Auto-COVID-VACC): Protocol for Multicenter Prospective Noninterventional Study
Source: JMIR Res Protoc. 2025 May 26;14:e60675. doi: 10.2196/60675 (PMC12149776; doi:10.2196/60675)
Supplement: Multimedia Appendix 3 [file resprot_v14i1e60675_app3.pdf]

Cologne, 2023/10/02

Primary endpoint definition for the Auto-COVID-VACC trial

Lutz Gieselmann, Veronica Di Cristanziano, Felix Dewald, Florian Klein

Background

Immunity against SARS-CoV-2 can be established through natural infection, vaccination, or a combination of both, which contributes to reducing the likelihood of reinfection and mitigating disease severity. A correlate of protection (CoP) is an immunological indicator that, when reaching a specific threshold, is associated with protective immunity against an infectious agent[1]. The identification of a CoP for SARS-CoV-2 has been a key objective since the beginning of the pandemic, particularly within the framework of COVID-19 vaccine trials[1–4]. A well-defined CoP would be instrumental in shaping vaccine strategies and public health measures aimed at limiting viral spread and disease burden. Additionally, a quantifiable CoP could assist healthcare providers in determining the necessity of booster vaccinations for individuals with immunocompromising conditions or other high-risk factors, particularly when antibody or neutralizing antibody (NAb) levels fall below a protective threshold.

Despite its importance, defining specific immune marker levels or protective thresholds as CoPs is a complex challenge. Multiple factors contribute to this complexity. Firstly, CoP identification is complicated by inter- and intra-study variability arising from population heterogeneity. Differences in immune marker levels are influenced by prior disease severity, time since infection or vaccination, patient demographics (e.g., age and comorbidities), assay methodologies (e.g., target antigens), circulating variants of concern (VOCs), and the specific vaccines administered[5–8]. Additionally, CoPs may vary across populations due to variations in genetic background, immune health, and previous exposures to SARS-CoV-2 or related coronaviruses. Moreover, many vaccine efficacy studies focus on preventing symptomatic disease rather than infection itself, meaning that while vaccines may demonstrate strong efficacy against severe disease, the CoP for infection prevention—particularly for asymptomatic cases—may differ.

Another challenge in defining a CoP is the lack of *Assay Standardization and Calibration*[9–12]. The ability to compare immune responses across studies is limited by the absence of universally accepted reference standards, particularly in observational research. To address this issue, the World Health Organization (WHO) has introduced the WHO International Standard for anti-SARS-CoV-2 immunoglobulins, advocating for the reporting of results in standardized units (binding antibody units per milliliter [BAU/mL]) for binding antibodies and international units per milliliter [IU/mL] for NAbs). However, broad adoption of these standards remains limited, complicating cross-study comparisons.

Cellular Immune Markers introduce additional complexity[13–15]. Many studies have not thoroughly investigated the potential role of cellular immunity markers as CoPs, despite the critical contributions of T cells and memory B cells to immune durability following infection or vaccination. This consideration is particularly significant given the continuous emergence of VOCs, which may compromise humoral immunity while cellular immunity offers sustained protection.

Additionally, the phenomenon of *Waning Immunity* further complicates CoP determination[7,16]. Over time, levels of immune markers such as neutralizing antibodies naturally decline. However, a reduction in these markers does not necessarily equate to a loss of protection, as memory B cells and T cells may still mediate an effective immune response upon re-exposure. This highlights the challenge of establishing a direct correlation between measurable immune markers and long-term protection.

Potential CoPs could encompass humoral, innate, cellular, or adaptive immune markers, which may be classified as either absolute or relative[2]. Absolute CoPs provide full protection at a specific threshold, whereas relative CoPs suggest that increasing marker levels correlate with enhanced protection or confer protection to a certain proportion of the population[1]. Further research is essential to refine our understanding of SARS-CoV-2 CoPs, facilitating the development of optimized vaccination policies and public health strategies to control the ongoing pandemic.

Objective:

The objective was to identify an absolute or relative CoP for the auto-COVID-VACC trial that is indicative for a decreased risk in mortality due to SARS-CoV-2 infection in response to an mRNA SARS-CoV-2 vaccine in patients post autologous stem cell transplantation.

Methods:

We conducted a targeted searching of relevant primary research articles and systemic reviews indexed on PubMed server from the years 2019 to 2023 that investigated immune markers as a potential CoP against SARS-CoV-2 infection[10,14,17–29]. We excluded preprint studies due to the lack of peer review. Key data extracted from eligible studies included: virus variants, measurement time point after vaccination, and immunity marker quantification such as serum binding (anti-Spike/RBD) or neutralizing activity.

Results:

After screening of PubMed database records, 15 studies were included in our review (see attached excel document)[10,14,17–29]. No studies reported a candidate immune marker representing an absolute (universal) CoP but rather suggested multiple humoral immune markers that may be applied as relative CoPs. These humoral immune markers comprised serum concentrations or titers of (1) anti-spike or RBD binding IgG[10,14,17,18,20–24,26–28] and/or (2) neutralizing antibodies[19,21,21–25,27]. The literature indicated that anti-spike IgG and NAb levels may be relative immune marker CoPs against SARS-CoV-2, with higher antibody levels being associated with a decreased risk of infection or disease severity. One recent study defined that the risk of death was inversely correlated to levels of spike IgG below the 20th percentile in a large cohort of 3,012 nursing homes residents (median age 86 years old)[29]. Study individuals were enrolled between September 2021 and August 2022 with follow-up of deaths to September 2022. The authors investigated longitudinal anti-spike IgG response after the 3rd and 4th booster vaccination. The authors define cases with an anti-spike antibody response lower than given percentile as low responders. Until the end of follow-up, 841 study subjects died. Out of them, 65 deaths occurred within 30 days after SARS-CoV-2 positive PCR. The 30-day mortality was higher in subjects with low levels of anti-spike IgG. In particular, individuals with the 20th lowest percentile of anti-spike IgG 60 days after the 3rd dose has a significantly increased risk of fatal COVID-19[29].

Based on these observations, we defined the 20th percentile of anti-Spike IgG as a cut-off value for the primary endpoint of the Auto-COVID-VACC study that may be indicative for a positive vaccine response[29]. To compute the corresponding cut-off value, we re-analyzed antibody levels of participants of a previously conducted seroprevalence study in patients of emergency departments with 1,411 patients in North Rhine-Westphalia (BESU study)[30]. We restricted our analysis to 1,140 participants that received no drug immunosuppression at the time of sampling. The 20th percentile of spike-IgG of those participants was 847 BAU/ml (**Figure 1**). The quantitative determination (BAU/ml) of IgG targeting the spike protein was performed using the chemiluminescence immunoassay (CLIA) LIAISON® SARS-CoV-2 Trimeric S IgG (Diasorin, Vicenza, Italy), which measures IgG against a recombinant trimeric spike antigen. Values ≥ 33.8 BAU/ml were interpreted as positive.

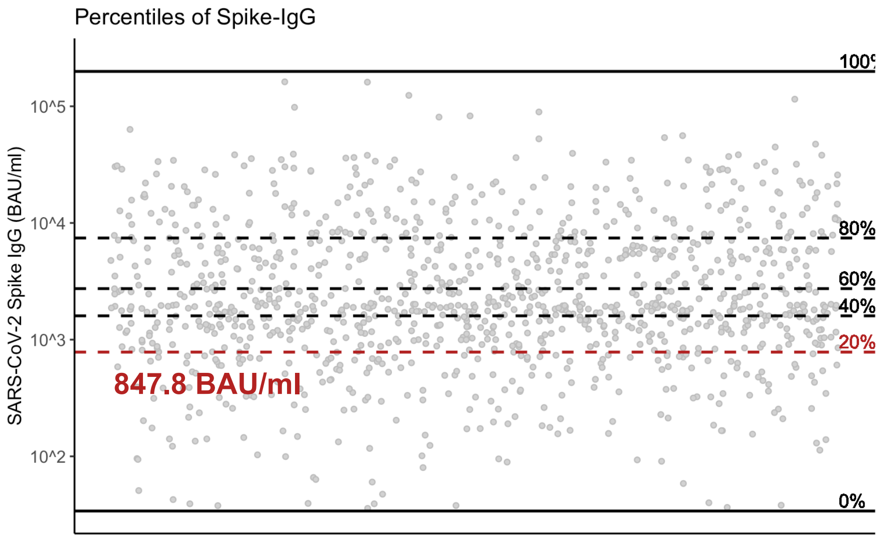

Figure 1: Percentiles of the spike-IgG levels of the analyzed serum samples of the BESU study.

Furthermore, we converted this spike-IgG cut-off to an RBD (receptor binding domain)-IgG cut-off. To this end, we analyzed 566 serum samples that were measured both in spike-IgG and RBD-IgG. Anti-RBD IgG were quantified by the chemiluminescent microparticle immunoassay (CMIA) SARS-CoV-2 IgG II Quant by Abbott. Our samples comprised 487 samples of the BESU study, 41 samples of hemodialysis patients after three and four doses of an mRNA vaccine, collected between September 2021 and April 2022, and 38 samples obtained in November 2022 from hemodialysis patients, most of them after bivalent booster vaccination. Applying linear robust regression, we modelled the anti-RBD IgG levels with the anti-spike IgG levels with the following equation (**Figure 2**):

$$\textit{Anti-RBD IgG} = \beta_0 + \beta_1 * \textit{anti-Spike IgG} + \varepsilon \qquad (1)$$

where both IgG levels are continuous variables. β_0 is the intercept and β_1 is the slope of the regression. ε represents the error term. Applying equation (1) to fit anti-RBD IgG, we predicted an anti-RBD IgG cut-off of 616 BAU/ml (CI: 562;670) corresponding to the above defined anti-spike IgG cut-off.

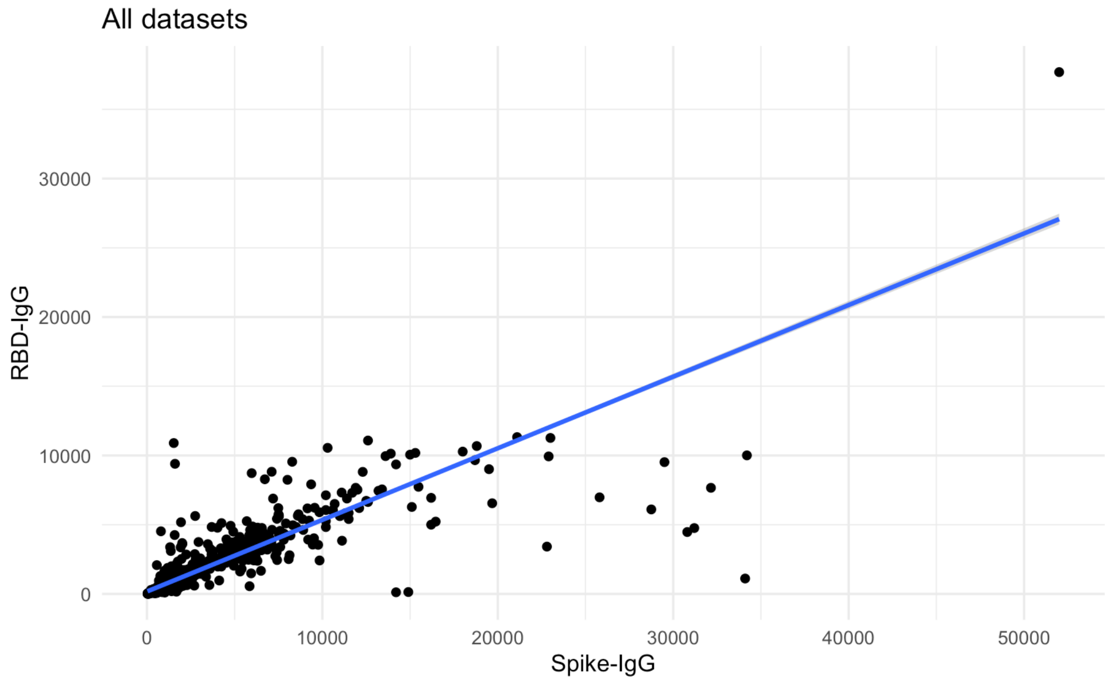

Figure 2: Robust linear regression as specified in equation 1.

Conclusions

Based on the present analysis, we suggest to define as non-responder to COVID-19 vaccine patients with an anti-spike IgG titer < 33.8 BAU/ml and as low responder patients with an anti-spike IgG titer < 847 BAU/ml (or < 616 anti-RBD IgG) at 8 weeks after vaccination.

Limitations

Our calculation of an anti-Spike IgG cut-off value for the AUTO-COVID-VACC study has several limitations. First, we transferred the 20th percentile as observed in longitudinal data by *Vikström et al.* to our seroprevalence data retrieved from a cross-sectional observation[29,30]. Thus, factors such as time since vaccination, number of vaccinations, or basic epidemiologic characteristics might differ between both cohorts. This potential mismatch might introduce inaccuracies into our computed cut-off value. Second, our cross-sectional data were collected from a convenience sample of patients of the emergency departments. While our measured seroprevalence aligns with that in random samples of the German population[31], we cannot generalize that the antibody levels that we have observed are representative of the entire German population or the population investigated by *Vikström et al.*. Third, our prediction of the anti-RBD-IgG is based on different heterogeneous data sets and populations of participants. Despite this heterogeneity, analyzing the data sets separately revealed comparable predictions. However, the use of diverse data sets introduces a potential source of variability in our results.

In summary, these limitations emphasize the need for caution in interpreting and generalizing our calculated cut-off values. Despite efforts to account for differences, the inherent disparities in study designs, participant characteristics, and sampling methods may affect the accuracy and broader applicability of our findings.

References

1. Gilbert, P.B., Donis, R.O., Koup, R.A., Fong, Y., Plotkin, S.A., and Follmann, D. (2022). A Covid-19 Milestone Attained — A Correlate of Protection for Vaccines. N Engl J Med *387*, 2203–2206. 10.1056/NEJMp2211314.

2. Krammer, F. (2021). A correlate of protection for SARS-CoV-2 vaccines is urgently needed. Nat Med *27*, 1147–1148. 10.1038/s41591-021-01432-4.

3. Baden, L.R., El Sahly, H.M., Essink, B., Kotloff, K., Frey, S., Novak, R., Diemert, D., Spector, S.A., Rouphael, N., Creech, C.B., et al. (2021). Efficacy and Safety of the mRNA-1273 SARS-CoV-2 Vaccine. N Engl J Med *384*, 403–416. 10.1056/NEJMoa2035389.

4. Voysey, M., Clemens, S.A.C., Madhi, S.A., Weckx, L.Y., Folegatti, P.M., Aley, P.K., Angus, B., Baillie, V.L., Barnabas, S.L., Bhorat, Q.E., et al. (2021). Safety and efficacy of the ChAdOx1 nCoV-19 vaccine (AZD1222) against SARS-CoV-2: an interim analysis of four randomised controlled trials in Brazil, South Africa, and the UK. The Lancet *397*, 99–111. 10.1016/S0140-6736(20)32661-1.

5. Misra, A., and Theel, E.S. (2022). Immunity to SARS-CoV-2: What Do We Know and Should We Be Testing for It? J Clin Microbiol *60*, e00482-21. 10.1128/jcm.00482-21.

6. Bertoletti, A., Le Bert, N., and Tan, A.T. (2022). SARS-CoV-2-specific T cells in the changing landscape of the COVID-19 pandemic. Immunity *55*, 1764–1778. 10.1016/j.immuni.2022.08.008.

7. Hamady, A., Lee, J., and Loboda, Z.A. (2022). Waning antibody responses in COVID-19: what can we learn from the analysis of other coronaviruses? Infection *50*, 11–25. 10.1007/s15010-021-01664-z.

8. Torres Ortiz, A., Fenn Torrente, F., Twigg, A., Hatcher, J., Saso, A., Lam, T., Johnson, M., Wagstaffe, H., Dhillon, R., Mai, A.L., et al. (2022). The influence of time on the sensitivity of SARS-CoV-2 serological testing. Sci Rep *12*, 10517. 10.1038/s41598-022-14351-2.

9. Zhu, F., Althaus, T., Tan, C.W., Costantini, A., Chia, W.N., Van Vinh Chau, N., Van Tan, L., Mattiuzzo, G., Rose, N.J., Voiglio, E., et al. (2022). WHO international standard for SARS-CoV-2 antibodies to determine markers of protection. The Lancet Microbe *3*, e81–e82. 10.1016/S2666-5247(21)00307-4.

10. Earle, K.A., Ambrosino, D.M., Fiore-Gartland, A., Goldblatt, D., Gilbert, P.B., Siber, G.R., Dull, P., and Plotkin, S.A. (2021). Evidence for antibody as a protective correlate for COVID-19 vaccines. Vaccine *39*, 4423–4428. 10.1016/j.vaccine.2021.05.063.

11. Knezevic, I., Mattiuzzo, G., Page, M., Minor, P., Griffiths, E., Nuebling, M., and Moorthy, V. (2022). WHO International Standard for evaluation of the antibody response to COVID-19 vaccines: call for urgent action by the scientific community. The Lancet Microbe *3*, e235–e240. 10.1016/S2666-5247(21)00266-4.

12. Kristiansen, P.A., Page, M., Bernasconi, V., Mattiuzzo, G., Dull, P., Makar, K., Plotkin, S., and Knezevic, I. (2021). WHO International Standard for anti-SARS-CoV-2 immunoglobulin. The Lancet *397*, 1347–1348. 10.1016/S0140-6736(21)00527-4.

13. Atti, A., Insalata, F., Carr, E.J., Otter, A.D., Castillo-Olivares, J., Wu, M., Harvey, R., Howell, M., Chan, A., Lyall, J., et al. (2022). Antibody correlates of protection from SARS-CoV-2 reinfection prior to vaccination: A nested case-control within the SIREN study. Journal of Infection *85*, 545–556. 10.1016/j.jinf.2022.09.004.

14. Barda, N., Canetti, M., Gilboa, M., Asraf, K., Indenboim, V., Weiss-Ottolenghi, Y., Amit, S., Zubli, D., Doolman, R., Mendelson, E., et al. (2023). The Association Between Prebooster Vaccination Antibody Levels and the Risk of Severe Acute Respiratory Syndrome Coronavirus 2 Infection. Clinical Infectious Diseases *76*, 1315–1317. 10.1093/cid/ciac886.

15. Dimeglio, C., Migueres, M., Bouzid, N., Chapuy-Regaud, S., Gernigon, C., Da-Silva, I., Porcheron, M., Martin-Blondel, G., Herin, F., and Izopet, J. (2022). Antibody Titers and Protection against Omicron (BA.1 and BA.2) SARS-CoV-2 Infection. Vaccines *10*, 1548. 10.3390/vaccines10091548.

16. Hernandez-Suarez, C., and Murillo-Zamora, E. (2022). Waning immunity to SARS-CoV-2 following vaccination or infection. Front. Med. *9*, 972083. 10.3389/fmed.2022.972083.

17. Yamamoto, S., Mizoue, T., and Ohmagari, N. (2023). Analysis of Previous Infection, Vaccinations, and Anti–SARS-CoV-2 Antibody Titers and Protection Against Infection With the SARS-CoV-2 Omicron BA.5 Variant. JAMA Netw Open *6*, e233370. 10.1001/jamanetworkopen.2023.3370.

18. Regev-Yochay, G., Lustig, Y., Joseph, G., Gilboa, M., Barda, N., Gens, I., Indenbaum, V., Halpern, O., Katz-Likvornik, S., Levin, T., et al. (2023). Correlates of protection against COVID-19 infection and intensity of symptomatic disease in vaccinated individuals exposed to SARS-CoV-2 in households in Israel (ICoFS): a prospective cohort study. The Lancet Microbe *4*, e309–e318. 10.1016/S2666-5247(23)00012-5.

19. Feng, S., Phillips, D.J., White, T., Sayal, H., Aley, P.K., Bibi, S., Dold, C., Fuskova, M., Gilbert, S.C., Hirsch, I., et al. (2021). Correlates of protection against symptomatic and asymptomatic SARS-CoV-2 infection. Nat Med *27*, 2032–2040. 10.1038/s41591-021-01540-1.

20. Roy, A., Saade, C., Josset, L., Clément, B., Morfin, F., Destras, G., Valette, M., Icard, V., Billaud, G., Oblette, A., et al. (2023). Determinants of protection against SARS-CoV-2 Omicron BA.1 and Delta infections in fully vaccinated outpatients. Journal of Medical Virology *95*, e28984. 10.1002/jmv.28984.

21. Gilboa, M., Gonen, T., Barda, N., Cohn, S., Indenbaum, V., Weiss-Ottolenghi, Y., Amit, S., Asraf, K., Joseph, G., Levin, T., et al. (2023). Factors Associated With Protection From SARS-CoV-2 Omicron Variant Infection and Disease Among Vaccinated Health Care Workers in Israel. JAMA Netw Open *6*, e2314757. 10.1001/jamanetworkopen.2023.14757.

22. Benkeser, D., Fong, Y., Janes, H.E., Kelly, E.J., Hirsch, I., Sproule, S., Stanley, A.M., Maaske, J., Villafana, T., Houchens, C.R., et al. (2023). Immune correlates analysis of a phase 3 trial of the AZD1222 (ChAdOx1 nCoV-19) vaccine. npj Vaccines *8*, 36. 10.1038/s41541-023-00630-0.

23. Fong, Y., Huang, Y., Benkeser, D., Carpp, L.N., Áñez, G., Woo, W., McGarry, A., Dunkle, L.M., Cho, I., Houchens, C.R., et al. (2023). Immune correlates analysis of the

PREVENT-19 COVID-19 vaccine efficacy clinical trial. Nat Commun *14*, 331. 10.1038/s41467-022-35768-3.

24. Gilbert, P.B., Montefiori, D.C., McDermott, A.B., Fong, Y., Benkeser, D., Deng, W., Zhou, H., Houchens, C.R., Martins, K., Jayashankar, L., et al. (2022). Immune correlates analysis of the mRNA-1273 COVID-19 vaccine efficacy clinical trial. Science *375*, 43–50. 10.1126/science.abm3425.

25. Fong, Y., McDermott, A.B., Benkeser, D., Roels, S., Stieh, D.J., Vandebosch, A., Le Gars, M., Van Roey, G.A., Houchens, C.R., Martins, K., et al. (2022). Immune correlates analysis of the ENSEMBLE single Ad26.COV2.S dose vaccine efficacy clinical trial. Nat Microbiol *7*, 1996–2010. 10.1038/s41564-022-01262-1.

26. Perez-Saez, J., Zaballa, M.-E., Lamour, J., Yerly, S., Dubos, R., Courvoisier, D.S., Villers, J., Balavoine, J.-F., Pittet, D., Kherad, O., et al. (2023). Long term anti-SARS-CoV-2 antibody kinetics and correlate of protection against Omicron BA.1/BA.2 infection. Nat Commun *14*, 3032. 10.1038/s41467-023-38744-7.

27. Falsey, A.R., Sobieszczyk, M.E., Hirsch, I., Sproule, S., Robb, M.L., Corey, L., Neuzil, K.M., Hahn, W., Hunt, J., Mulligan, M.J., et al. (2021). Phase 3 Safety and Efficacy of AZD1222 (ChAdOx1 nCoV-19) Covid-19 Vaccine. N Engl J Med *385*, 2348–2360. 10.1056/NEJMoa2105290.

28. Wei, J., Matthews, P.C., Stoesser, N., Newton, J.N., Diamond, I., Studley, R., Taylor, N., Bell, J.I., Farrar, J., Kolenchery, J., et al. (2023). Protection against SARS-CoV-2 Omicron BA.4/5 variant following booster vaccination or breakthrough infection in the UK. Nat Commun *14*, 2799. 10.1038/s41467-023-38275-1.

29. Vikström, L., Fjällström, P., Gwon, Y.-D., Sheward, D.J., Wigren-Byström, J., Evander, M., Bladh, O., Widerström, M., Molnar, C., Rasmussen, G., et al. (2023). Vaccine-induced correlate of protection against fatal COVID-19 in older and frail adults during waves of neutralization-resistant variants of concern: an observational study. The Lancet Regional Health - Europe *30*, 100646. 10.1016/j.lanepe.2023.100646.

30. Dewald, F., Pirkl, M., Paluschinski, M., Kühn, J., Elsner, C., Schulte, B., Knüfer, J., Ahmadov, E., Schlotz, M., Oral, G., et al. (2023). Impaired humoral immunity to BQ.1.1 in convalescent and vaccinated patients. Nat Commun *14*, 2835. 10.1038/s41467-023-38127-y.

31. Schulze-Wundling, K., Ottensmeyer, P.F., Meyer-Schlinkmann, K.M., Deckena, M., Krüger, S., Schlinkert, S., Budde, A., Münstermann, D., Töpfner, N., Petersmann, A., et al. (2023). Immunity against SARS-CoV-2 in the German population. Deutsches Ärzteblatt international. 10.3238/arztebl.m2023.0072.

Appendix I: List of primary research articles and and systemic reviews indexed on PubMed server from the years 2019 to 2023

Appendix II: Dewald, F., Pirkl, M., Paluschinski, M., Kühn, J., Elsner, C., Schulte, B., Knüfer, J., Ahmadov, E., Schlotz, M., Oral, G., et al. (2023). Impaired humoral immunity to BQ.1.1 in convalescent and vaccinated patients. Nat Commun *14*, 2835. 10.1038/s41467-023-38127-y.
